# Stemness-Suppressive Effect of Bibenzyl from *Dendrobium ellipsophyllum* in Human Lung Cancer Stem-Like Cells

**DOI:** 10.1155/2021/5516655

**Published:** 2021-07-23

**Authors:** Pornchanok Taweecheep, Hnin Ei Ei Khine, Anirut Hlosrichok, Gea Abigail Uy Ecoy, Boonchoo Sritularak, Eakachai Prompetchara, Pithi Chanvorachote, Chatchai Chaotham

**Affiliations:** ^1^Department of Biochemistry and Microbiology, Faculty of Pharmaceutical Sciences, Chulalongkorn University, Bangkok 10330, Thailand; ^2^Department of Pharmacy, School of Health Care Professions, University of San Carlos, 6000 Cebu, Philippines; ^3^Department of Pharmacognosy and Pharmaceutical Botany, Faculty of Pharmaceutical Sciences, Chulalongkorn University, Bangkok 10330, Thailand; ^4^Department of Laboratory Medicine, Faculty of Medicine, Chulalongkorn University, Bangkok 10330, Thailand; ^5^Center of Excellence in Vaccine Research and Development (Chula Vaccine Research Center-Chula VRC), Faculty of Medicine, Chulalongkorn University, Bangkok 10330, Thailand; ^6^Department of Pharmacology and Physiology, Faculty of Pharmaceutical Sciences, Chulalongkorn University, Bangkok 10330, Thailand; ^7^Cell-Based Drug and Health Products Development Research Unit, Faculty of Pharmaceutical Sciences, Chulalongkorn University, Bangkok 10330, Thailand

## Abstract

Cancer stem-like cells (CSCs) are key mediators driving tumor initiation, metastasis, therapeutic failure, and subsequent cancer relapse. Thus, targeting CSCs has recently emerged as a potential strategy to improve chemotherapy. In this study, the anticancer activity and stemness-regulating capacity of 4,5,4′-trihydroxy-3,3′-dimethoxybibenzyl (TDB), a bibenzyl extracted from *Dendrobium ellipsophyllum*, are revealed in CSCs of various human lung cancer cells. Culture with TDB (5–10 *μ*M) strongly abolished tumor-initiating cells in lung cancer H460, H23, and A549 cells in both anchorage-dependent and anchorage-independent colony formation assays. Through the 3D single-spheroid formation model, attenuation of self-renewal capacity was observed in CSC-enriched populations treated with 1–10 *μ*M TDB for 7 days. Flow cytometry analysis confirmed the attenuation of %cell overexpressing CD133, a CSC biomarker, in TDB-treated lung cancer spheroids. TDB at 5–10 *μ*M remarkably suppressed regulatory signals of p-Akt/Akt, p-GSK3*β*/GSK3*β*, and *β*-catenin corresponding to the downregulated mRNA level of stemness transcription factors including Nanog, Oct4, and Sox2. Moreover, the antiapoptosis Bcl-2 and Mcl-1 proteins, which are downstream molecules of Akt signaling, were evidently decreased in CSC-enriched spheroids after culture with TDB (1–10 *μ*M) for 24 h. Interestingly, the diminution of Akt expression by specific siAkt effectively reversed suppressive activity of TDB targeting on the CSC phenotype in human lung cancer cells. These findings provide promising evidence of the inhibitory effect of TDB against lung CSCs via suppression of Akt/GSK3*β*/*β*-catenin cascade and related proteins, which would facilitate the development of this bibenzyl natural compound as a novel CSC-targeted therapeutic approach for lung cancer treatment.

## 1. Introduction

Lung cancer has been recognized as one of the most common cancers worldwide, accounting for a large proportion of cancer deaths [1]. Comprising approximately 80% of all cases, non-small-cell lung cancer (NSCLC) is the predominant type, the majority of which are diagnosed at an advanced or metastatic stage [2]. Despite the progress in cancer research, there is still a low five-year survival rate among lung cancer patients [3, 4]. Recurrence of tumor lesions resulting in high mortality rates indicates a failure of available therapeutic regimens for lung cancer [5, 6]. Chemotherapy, especially when combined with other remedies such as surgery or radiation, has been shown to offer a survival advantage to NSCLC patients [7, 8]. However, key barriers in chemotherapy such as innate or developed chemoresistance and high incidence of cancer relapse after completion of therapeutic regimens have been consistently reported [9, 10].

It has been accepted that the heterogeneity of cancer population crucially influences the relatively low success rate of current anticancer drugs [11]. The subpopulation of cancer stem-like cells (CSCs) within tumor tissue is imbued with stemness features of self-renewal and multilineage differentiation, thus allowing tumor initiation from the remaining CSCs after chemotherapy [12]. CSCs that highly express CD133 (prominin-1) on cellular membrane isolated from both clinical specimens of lung cancer patients and human lung cancer cells have been documented to be mediators of drug resistance and tumor initiation properties [13, 14]. Conventional chemotherapeutic agents, however, do not eradicate CSC subpopulations [15]. Therefore, novel chemotherapeutic moieties targeting CSCs are urgently needed for improved lung cancer treatment [16].

The stemness phenotypes of CSCs are mediated by several pluripotent transcription factors including Oct4 (octamer-binding transcription factor 4), Sox2 ((sex determining region Y)-box 2), and Nanog [17, 18]. Oct4 plays a critical role in the tumorigenesis, self-renewal, and pluripotency of CSCs obtained from different cancers [19, 20]. Likewise, Sox2 is an essential mediator of self-renewal in embryonic stem cells (ESCs) [21]. The overexpression of Oct4 and Sox2 influences not only the differentiation of mesenchymal stem cells but also the tumor-initiating capacity of CSCs [22]. Additionally, Oct4 and Sox2 transcription factors responsible for self-renewal and pluripotency are found to mediate such activities through complex interactions with Nanog [23]. Accumulating evidence presents the reduction of Oct4, Sox2, and Nanog expression levels as an effective strategy to suppress CSC characteristics in lung cancer [24–26]. The modulation of CSC features and related proteins is also regulated by upstream signaling of PI3K (phosphoinositide 3-kinases)/Akt (protein kinase B)/*β*-catenin pathway [27, 28]. Activation of Akt with consequent phosphorylation of GSK3*β* (glycogen synthase kinase 3*β*) results in the release of *β*-catenin from the GSK3*β* degradation complex [29]. The augmented level of *β*-catenin in turn stimulates the transcription of Oct4, Sox2, and Nanog [30]. Thus, the inhibition on Akt/GSK3*β* pathway efficiently decreases CD133^high^-expressing cells and tumor-initiating activity in lung cancer [31]. Interestingly, various natural compounds have demonstrated anticancer activity, especially on CSCs via suppression of Akt-mediated stemness regulatory proteins [26, 32].

Bibenzyl of 4,5,4′-trihydroxy-3,3′-dimethoxybibenzyl (TDB) extracted from *Dendrobium ellipsophyllum* has gained interest due to its induction of apoptosis and repression of epithelial to mesenchymal transition (EMT) through Akt inhibition in human lung cancer cells [33]. Since targeting Akt-related signal is a promising strategy for CSC-directed therapy in lung cancer [25], the previous findings on TDB have prompted further interest in the potential of this natural bibenzyl to target lung CSCs, a facet which has not yet been explored. This study aimed to evaluate the suppressive effect of TDB and the relevant underlying mechanisms on CSCs in human lung cancer cells. The obtained information would support the further development of the bibenzyl from *D. ellipsophyllum* as a potential chemotherapeutic drug using a CSC-targeted approach for lung cancer therapy.

## 2. Materials and Methods

### 2.1. Chemical Reagents

TDB was extracted from *D. ellipsophyllum* as previously described [33]. Hoechst33342, propidium iodide (PI), 3-(4,5-dimethylthiazol-2-yl)-2,5-diphenyltetrazolium bromide (MTT), dimethyl sulfoxide (DMSO), crystal violet solution (1% w/v), formaldehyde solution (37% w/v), and skim milk powder were purchased from Sigma Chemical, Inc. (St. Louis, MO, USA). Primary antibodies specific to Akt, p-Akt (Ser 473), GSK3*β*, p-GSK3*β* (Ser 9), *β*-catenin, Bcl-2 (B-cell lymphoma 2), Mcl-1 (myeloid cell leukemia 1), GAPDH (glyceraldehyde 3-phosphate dehydrogenase), and horseradish peroxidase (HRP) labeled secondary antibody were obtained from Cell Signaling Technology (Danvers, MA, USA). Rabbit anti-CD133 antibody and Alexa Fluor 488-conjugated goat anti-rabbit IgG were procured from Thermo Scientific (Rockford, IL, USA). Immobilon Western chemiluminescent HRP substrate was obtained from Millipore (Billerica, MA, USA).

### 2.2. Cell Culture

Human non-small lung cancer H460, H23, and A549 cells were sourced from American Type Culture Collection (Manassas, VA, USA). Roswell Park Memorial Institute (RPMI) 1640 medium (Gibco, Gaithersburg, MA, USA) was used to maintain H460 and H23 cells, whereas A549 cells were cultured in Dulbecco's modified eagle (DMEM) medium (Gibco, Gaithersburg, MA, USA). Human dermal papilla cells (DPCs) obtained from Applied Biological Materials Inc. (Richmond, BC, Canada) were cultured in Prigrow III medium (Applied Biological Materials Inc., Richmond, BC, Canada). All culture media were supplemented with 10% fetal bovine serum (FBS), 2 mmol/L of l-glutamine, and 100 units/mL of penicillin/streptomycin (Gibco, Gaithersburg, MA, USA). The cells were maintained in an incubator at 37°C under a humidified atmosphere of 5% CO_2_ until reaching 70–80% confluence for use in experiments.

### 2.3. Cytotoxicity Assay

Cell viability was examined by MTT colorimetric assay. Firstly, cells seeded at a density of 1 × 10^4^ cells/well in a 96-well plate were cultured with TDB (0–300 *μ*M) for 24 h. Then, the cells were incubated with MTT (0.4 mg/mL) for 3 h at 37°C in a dark place. The supernatant was removed before adding 100 *μ*L/well of DMSO to dissolve purple formazan crystal. The intensity of the formazan product was determined via spectrophotometry at 570 nm using a microplate reader (Anthros, Durham, NC, USA). The percentage (%) of cell viability was calculated from the absorbance ratio between treated and untreated control cells.

### 2.4. Nuclear Staining Assay

Mode of cell death was evaluated through nuclear costaining with Hoechst33342 and PI. The cells were seeded at a density of 1 × 10^4^ cells/well in a 96-well plate for 12 h and then further treated with TDB (0–50 *μ*M) for 24 h. After the incubation with Hoechst33342 (10 *μ*g/mL)/PI (5 *μ*g/mL) solution at 37°C for 30 min, apoptosis cells characterized by bright blue fluorescence of Hoechst33342 and PI-positive necrosis cells presenting red fluorescence were visualized and counted under a fluorescence microscope (Olympus IX51 with DP70, Olympus, Japan).

### 2.5. Spheroid Formation Assay

The three-dimensional (3D) spheroid formation was carried out in culture media supplemented with 1% FBS in an ultralow attachment plate for the enrichment of CSC populations in cancer colony [34]. Briefly, cells were grown in a 6-well ultralow attachment plate at a density of 3 × 10^4^ cells/well and 500 *μ*L of 1% FBS supplemented media was added to maintain the cells every 3 days for 7 days. After repeating 2 cycles of the described anchorage-independent growth, the secondary spheroids were transferred into 24-well ultralow attachment plate as single colonies. CSC-enriched spheroids were treated with various concentrations of TDB and further incubated for 7 days. At days 0, 1, 3, 5, and 7, the spheroids were photographed using an inverted microscope (Nikon Ts2, Nikon, Japan). The spheroid size at the observation day was calculated relative to the size at day 0 and presented as relative spheroid size.

### 2.6. Detection of CD133 via Flow Cytometry

The secondary CSC spheroids grown in media supplemented with 1% FBS with or without TDB were subjected to flow cytometry for evaluation of expression level of a CSC protein marker, CD133, on cell membrane. After 3 days of incubation, CSC-enriched spheroids were harvested by centrifugation at 200 g for 4 min. The cells were washed twice with PBS (pH 7.4) and resuspended in PBS containing 10% FBS (incubation buffer) as single-cell suspension. Next, the cell suspension was further incubated on ice with an anti-CD133 antibody for 1 h. After removal of primary antibody and washing with the incubation buffer, Alexa Fluor 488-conjugated secondary antibody was added, and the cells were incubated on ice for another 30 min. The labeled cells were washed with the incubation buffer and further resuspended in PBS for measurement of CD133-associated fluorescence intensity via Guava easyCyte flow cytometer using InCyte 3.3 software (EMD Millipore, Billerica, MA, USA).

### 2.7. Limiting Dilution Assay

In this study, a limiting dilution assay (LDA) was used to determine the effect of TDB on CSC populations possessing tumor-initiating capability [35]. Briefly, the cells were prepared in media supplemented with 1% FBS and seeded in a 96-well ultralow attachment plate at a density of 1, 10, 50, 100, and 200 cells/well. Then, the cells were cultured with or without different concentrations of TDB for 14 days. The number of colonies formed was counted and photographed under an inverted microscope (Nikon Ts2, Nikon, Japan).

### 2.8. Clonogenic Assay

The ability of cancer cells to proliferate and generate tumor colonies after treatment with TDB was also evaluated through clonogenic or colony formation assay [36]. Human lung cancer cells at a density of 1 × 10^5^ cells/well in the 6-well plate were cultured with various concentrations of TDB for 24 h. After removing undetached cells by washing with PBS, the remaining cells were made into single-cell suspension. Next, the viable cells derived after TDB treatment were seeded at a density of 250 cells/well in the 6-well plate and cultured for 7 days. The formed colonies were then counted after fixing with methanol and acetic acid (ratio 3 : 1) solution followed by staining with 0.05% w/v crystal violet in 4% formaldehyde.

### 2.9. Western Blotting

The secondary CSC-enriched spheroids were treated with different concentrations of TDB for 24 h. After centrifugation, the treated spheroids were incubated with 1× radioimmunoprecipitation assay (RIPA) lysis buffer (Thermo Scientific, Rockford, IL, USA) supplemented with a freshly prepared protease inhibitor cocktail (Roche Applied Science, Indianapolis, IN, USA) on ice for 45 min. The supernatant was collected for the determination of total protein content using bicinchoninic acid (BCA) protein assay kit (Pierce Biotechnology, Rockford, IL, USA). Equal amounts of protein were separated through 10% sodium dodecyl sulfate-polyacrylamide gel electrophoresis (SDS-PAGE). The proteins were then transferred onto 0.45 *μ*m nitrocellulose membranes (Bio-Rad Laboratories, Hercules, CA, USA), which in turn was immersed in 5% nonfat dry milk in TBST (25 mmol/L Tris-HCl, pH 7.4, 125 mmol/L NaCl, 0.1% Tween 20) at room temperature for 45 min. The membranes were then incubated with specific primary antibodies at 4°C overnight. After washing with TBST for 5 min × 3 times, the membranes were further probed with HRP-conjugated secondary antibody for 2 h at 25°C. A chemiluminescent reaction with Immobilon Western chemiluminescent HRP substrate was used to detect the signals of specific proteins, which was quantified via analyst/PC densitometry software (Bio-Rad Laboratories, Hercules, CA, USA).

### 2.10. Reverse Transcription Quantitative Real-Time PCR (RT-qPCR)

Total RNA was extracted from CSC-enriched spheroids after treatment with different concentrations of TDB for 24 h. RNA samples were reverse transcribed into cDNA using RevertAid First Strand cDNA Synthesis Kit (Thermo Scientific, Rockford, IL, USA). cDNA was then quantified by measuring the absorbance at 260 nm using Thermo Scientific NanoDrop One microvolume UV-Vis spectrophotometers (Thermo Scientific, Rockford, IL, USA). The expression levels of transcription factor genes (Nanog, Oct4, and Sox2) and housekeeping gene GAPDH in the spheroids were analyzed by reverse transcription quantitative real-time PCR (RT-qPCR) using the CFX 96 Real-Time PCR system (Bio-Rad Laboratories, Hercules, CA, USA). The RT-qPCR was performed using Luna Universal qPCR Master Mix (Bio-Rad Laboratories, Hercules, CA, USA). The designed primers used in this study are presented as follows:

  Nanog: forward: 5′-ACCAGTCCCAAAGGCAAACA-3′
     reverse: 5′-TCTGCTGGAGGCTGAGGTAT-3′
  Oct-4: forward: 5′-AAGCGATCAAGCAGCGACTA-3′
     reverse: 5′-GAGACAGGGGGAAAGGCTTC-3′
  Sox2: forward: 5′-ACATGAACGGCTGGAGCAA-3′
     reverse: 5′-GTAGGACATGCTGTAGGTGGG-3′
  GAPDH: forward: 5′-GACCACAGTCCATGCCATCA-3′
     reverse: 5′-CCGTTCAGCTCAGGGATGAC-3′.

The initial denaturation step was performed at 95°C for 3 min, followed by 40 cycles of denaturation at 95°C for 5 sec and primer annealing at 57°C for 30 sec. Analysis was performed using the comparative Cq value method. The relative expression of each gene was normalized against the housekeeping gene product.

### 2.11. siRNA Transfection

Lung cancer H460 cells were transfected with either small interfering RNA (siRNA) specific to human Akt mRNA (siAkt) or si-mismatch control (siCtrl) using Lipofectamine 2000^®^ (Invitrogen; Thermo Fisher Scientific, Waltham, MA, USA) as recommended by the manufacturer's protocol. The synthesized siRNAs used in this study are presented as follows:

  siAkt: sense: 5′-GGAGAUCAUGCAGCAUCGC-3′
     antisense: 5′-GCGAUGCUGCAUGAUCUCC-3′
  siCtrl: sense: 5′-GGGAAUCAUAAAGCAUUUC-3′
     antisense: 5′-CCGGGGCUGCAUAAACUUC-3′.

Briefly, 100 nM siRNAs in OptiMEM cell culture medium (Thermo Fisher Scientific, Waltham, MA, USA) were incubated with Lipofectamine 2000^®^ mixture for 15 min at room temperature and then added to lung cancer H460 cells. After incubation at 37°C for 48 h, the transfected cells were further subjected to 3D spheroid formation, LDA, and clonogenic assay for evaluating the anticancer activity targeting CSCs of TDB. The silencing of Akt and p-Akt protein expression level was evaluated by Western blot analysis.

### 2.12. Statistical Analysis

The data from three independent experiments are presented as means ± standard deviation (SD). Using SPSS statistical software version 22 (IBM Corp., Armonk, NY, USA), one-way analysis of variance and Tukey HSD post hoc test were performed, with statistical significance at *p* value < 0.05.

## 3. Results

### 3.1. Cytotoxic Profile of TDB in Human Lung Cancer Cells

**Figure 1:**
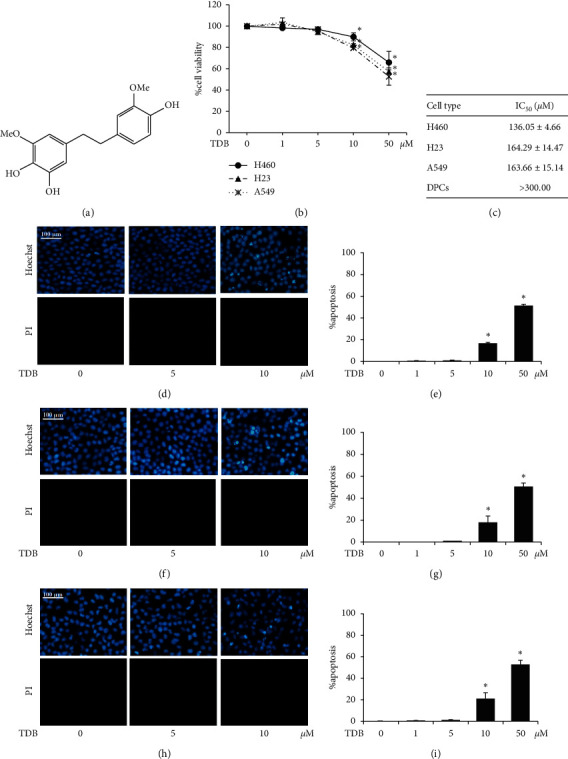
Cytotoxicity profile of TDB extracted from *Dendrobium ellipsophyllum* in human lung cancer cells. (a) Molecular structure of TDB (4,5,4′-trihydroxy-3,3′-dimethoxybibenzyl). (b) Cell viability in human lung cancer H460, H23, and A549 cells after treatment with 0–50 *μ*M TDB for 24 h was determined by the MTT assay. (c) Selective anticancer activity of TDB against human lung cancer cells was evidenced by approximately 2-fold higher than the half-maximum inhibitory concentration (IC_50_) in human dermal papilla cells (DPCs) compared with various lung cancer cells. The nuclear staining assay obviously showed bright blue fluorescence of Hoechst33342, which represents condensed chromatin and/or fragmented nuclei signifying apoptosis in (d) H460, (f) H23, and (h) A549 cells cultured with TDB at 10 *μ*M. Meanwhile, there was no detectable necrosis in all treated lung cancer cells as indicated by the absence of red fluorescence from propidium iodide (PI) staining. Percent apoptosis was also calculated in TDB-treated (e) H460, (g) H23, and (i) A549 cells costained with Hoechst33342/PI. Values are means of the independent triplicate experiments ± SD. ^*∗*^*p* < 0.05 versus nontreated control cells.

MTT viability assay was used to evaluate the toxicity of TDB (Figure 1(a)) in human non-small lung cancer cells. After 24 h of treatment at 0–50 *μ*M, TDB reduced viability in human lung cancer H460, H23, and A549 cells in a dose-dependent manner (Figure 1(b)). The relatively nontoxic effect of TDB was noted at 1–5 *μ*M, whereas a significant reduction of %cell viability was observed at 10–50 *μ*M compared with the untreated control cells. Because hair loss is one of the serious side effects induced by chemotherapeutic agents [37, 38], the determination of half-maximum inhibitory concentration (IC_50_) was additionally performed in both lung cancer cells and human dermal papilla cells (DPCs) to verify the safety profile of TDB. As indicated in Figure 1(c), the selective cytotoxicity of TDB against human non-small lung cancer cells was evidenced with IC_50_ value at higher than 300 *μ*M in DPCs, whereas lung cancer H460, H23, and A549 cells possessed IC_50_ of TDB approximately at 136.05 ± 4.66, 164.29 ± 14.47, and 163.66 ± 15.14 *μ*M, respectively.

To further clarify cytotoxic activity, TDB-treated lung cancer cells were costained with Hoechst33342/PI for detection of the mode of cell death. Apoptosis cells characterized by condensed chromatin and/or fragmented nuclei, which was indicated by bright blue fluorescence of Hoechst33342, were clearly observed in H460, H23, and A549 cells in response to 10 *μ*M TDB treatment (Figures 1(d), 1(f), and 1(h), respectively). It should be noted that there were no necrosis cells stained with red fluorescence of PI in TDB-treated lung cancer cells. Corresponding to the cell viability results, the augmentation of %apoptosis was significantly noted in all lung cancer cells treated with TDB at a concentration of 10–50 *μ*M (Figures 1(e), 1(g), and 1(i)).

### 3.2. Diminution of Tumor-Initiating Activity in TDB-Treated Lung Cancer Cells

**Figure 2:**
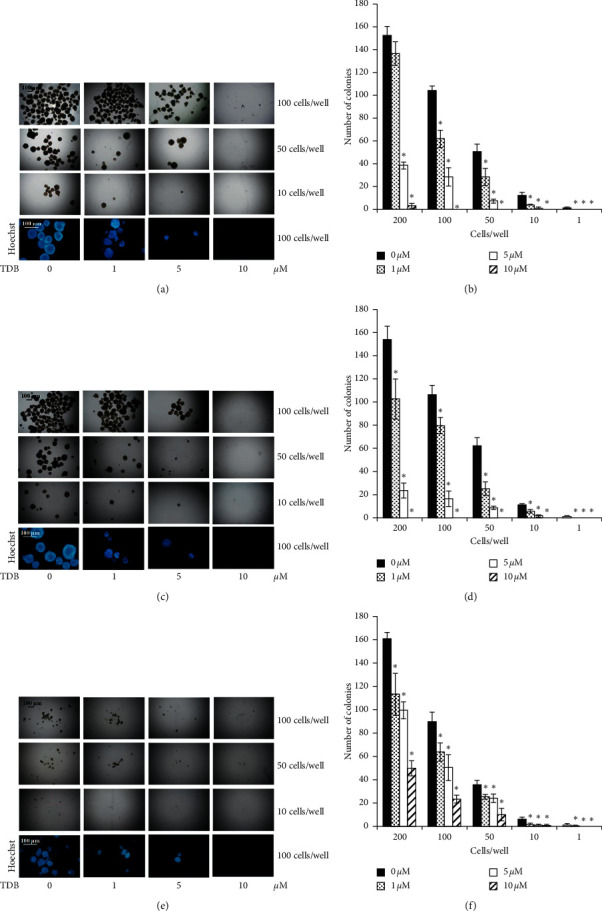
Inhibitory effect of TDB on tumor-initiating subpopulations in human lung cancer cells observed through limiting dilution assay (LDA). The quantities of tumor-initiating cells in (a) H460, (c) H23, and (e) A549 cells notably decreased in a dose-dependent manner after incubation with 1–10 *μ*M TDB for 14 days. Photographs of tumor colonies were obtained through optical microscopy (4x), while images with bright blue fluorescence of Hoechst33342 were obtained by fluorescence microscopy (10x) at day 14 of treatment. The dramatic decrease in the colony number correlated with the findings of no observable Hoechst33342-stained colonies in (b) H460, (d) H23, and (f) A549 cells cultured with 10 *μ*M TDB. Values are means of the independent triplicate experiments ± SD. ^*∗*^*p* < 0.05 versus nontreated control.

To evaluate the inhibitory effect of TDB on the frequency of tumor-initiating cells in cancer populations, the limiting dilution assay (LDA) was carried out in human lung cancer cells at various cell densities (1, 10, 50, 100, and 200 cells/well) [39]. The presence of tumor-initiating cells in human lung cancer H460, H23, and A549 cells was clearly indicated by the generation of cancer colonies under detachment condition of LDA at all cell densities (Figures 2(a), 2(c), and 2(e), respectively). Interestingly, the population of tumor-initiating cells in H460, H23, and A549 cells was significantly diminished in a dose-dependent manner after incubation with 1–10 *μ*M TDB as presented in Figures 2(b), 2(d), and 2(f), respectively. Strong suppression of tumor initiation was indicated by low colony number and absence of Hoechst33342-stained colonies in response to treatment of TDB at 10 *μ*M, especially in lung cancer H460 and H23 cells.

**Figure 3:**
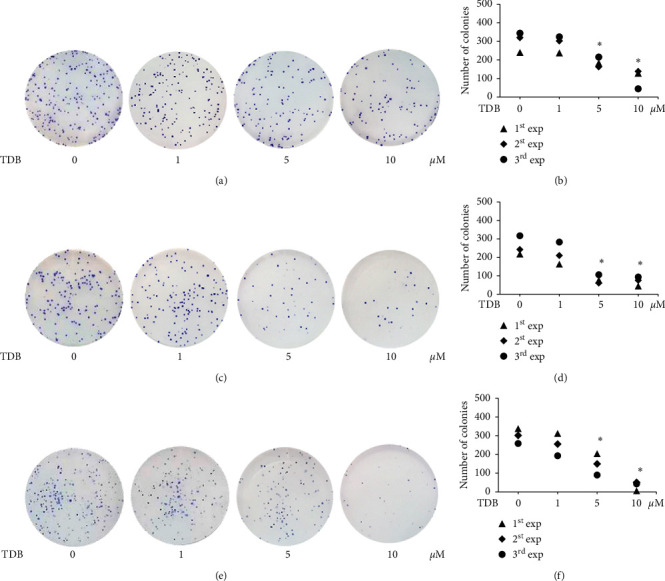
Clonogenic assay demonstrates the inhibitory effect of TDB on cancer colony formation. After treatment with 1–10 *μ*M of TDB for 24 h, the derived human lung cancer (a) H460, (c) H23, and (e) A549 cells were further cultured for 7 days and then stained with crystal violet for determination of colony formation capacity compared to nontreated cells. A significant reduction of the colony number obtained from an independent experiment (exp) was demonstrated in human lung cancer (b) H460, (d) H23, and (f) A549 cells treated with TDB at 5–10 *μ*M. ^*∗*^*p* < 0.05 versus nontreated control.

The clonogenic or colony formation assay is based on the capacity of a single cancer cell to regenerate a cancer colony. It is widely used for the determination of the effectiveness of cytotoxic compounds to eliminate proliferating tumor cells [40, 41]. After culture for 7 days, crystal violet-stained cancer colonies initiated from single cells of H460, H23, and A549 were evaluated, and the representative images are sequentially presented in Figures 3(a), 3(c), and 3(e). Human lung cancer cells that were derived after culture with TDB (1–10 *μ*M) for 24 h possessed lower capacity of colony formation compared with nontreated cells. The dramatic reduction of colony number was correspondingly observed in lung cancer H460, H23, and A549 cells treated with 5–10 *μ*M TDB (Figures 3(b), 3(d), and 3(f), respectively).

Taken together, these results reveal that TDB could restrain the tumor-initiating capacity of human lung cancer cells in both anchorage-independent and anchorage-dependent colony formation models. It is worth noting that the diminution on tumor-initiating cells was promptly observed at a nontoxic (5 *μ*M) concentration of TDB.

### 3.3. Suppressive Effect of TDB on CSC-Enriched Spheroids of Human Lung Cancer Cells

**Figure 4:**
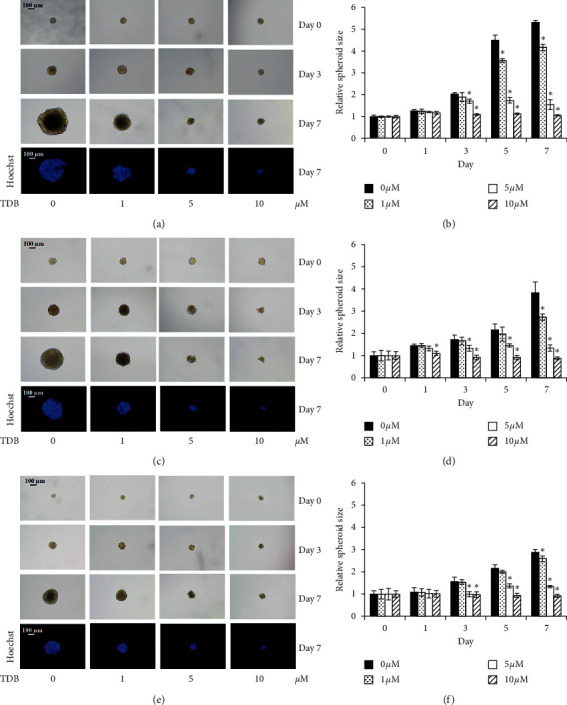
Evaluation of the self-renewal property of CSC-enriched populations. Single secondary spheroids of human lung cancer (a) H460, (c) H23, and (e) A549 cells treated with 1–10 *μ*M of TDB were visibly smaller than the nontreated control. At day 7 of treatment, all single 3D spheroids were visualized by optical microscopy (10x), and images depicting bright blue fluorescence of Hoechst33342 were taken by fluorescence microscopy (10x). Relative to the control group, the sizes of CSC-enriched spheroids of lung cancer (b) H460, (d) H23, and (f) A549 cells were significantly reduced after treatment of TDB at 5–10 *μ*M for 3–7 days. Values are means of the independent triplicate experiments ± SD. ^*∗*^*p* < 0.05 versus nontreated spheroids.

Three-dimensional (3D) spheroid formation or colonosphere assay is commonly used to evaluate the self-renewal feature of CSCs [42]. Herein, CSC-targeted anticancer activity of TDB was evaluated in secondary CSC spheroids of human lung cancer cells. Although Hoechst33342 nuclear staining showed no detectable cell death in CSC-enriched spheroids after culture with TDB (1–10 *μ*M) for 7 days, TDB-treated spheroids of lung cancer H460, H23, and A549 cells were markedly smaller than untreated control counterparts (Figures 4(a), 4(c), and 4(e), respectively). Indeed, the reduced relative size of CSC spheroids was noted early on day 3 of the incubation with 5–10 *μ*M TDB (Figures 4(b), 4(d), and 4(f)).

### 3.4. TDB Decreases CD133^high^-Expressing Cells in Lung CSC-Enriched Spheroids

**Figure 5:**
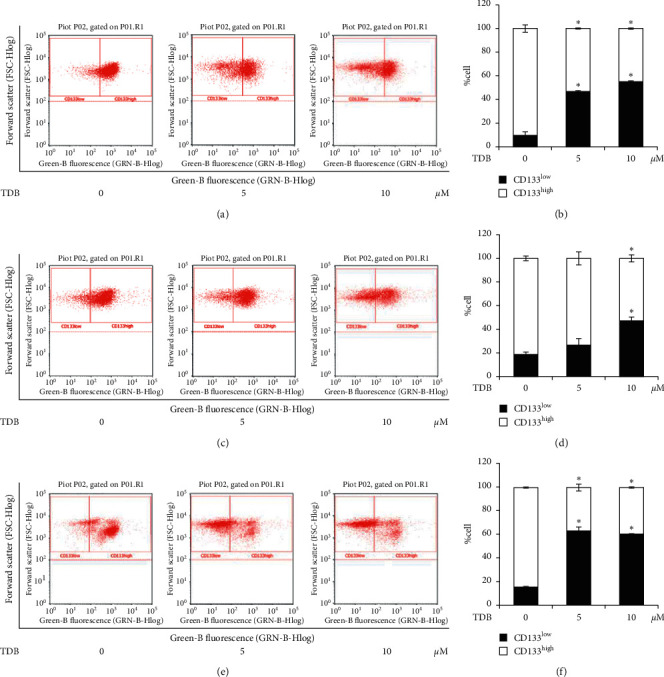
Flow cytometry analysis of CD133 expression in CSC-enriched spheroids of human lung cancer. The dot plots obtained from flow cytometry obviously showed the alteration of CD133-overexpressing subpopulations in lung cancer (a) H460, (c) H23, and (e) A549 spheroids treated with 5–10 *μ*M TDB. The significant reduction of %CD133^high^-expressing cell correlated with augmentation of the CD133^low^ subpopulation in TDB-treated (b) H460, (d) H23, and (f) A549 spheroids. Values are means of the independent triplicate experiments ± SD. ^*∗*^*p* < 0.05 versus nontreated spheroids.

Because CD133 is considered as a surface marker for lung CSC detection [43], the expression of CD133 was investigated via flow cytometry in H460, H23, and A549 secondary spheroids cultured with TDB for 3 days based on the significant alteration of relative size resulting from this treatment condition. Flow cytometry plots distinctly demonstrate that TDB (5–10 *μ*M) could diminish the CD133^high^ subpopulations in lung cancer H460, H23, and A549 spheroids as separately presented in Figures 5(a), 5(c), and 5(e). CSC-enriched spheroids of lung cancer H460, H23, and A549 cells comprised approximately 80% of CD133^high^-expressing cells, whereas the major subpopulation in TDB-treated spheroids consisted of CD133^low^-expressing lung cancer cells (Figures 5(b), 5(d), and 5(f), respectively). The augmentation of CD133^low^-expressing cells corresponds to the diminution of relative CSC spheroid size after TDB treatment (Figure 4). These results suggest that TDB inhibits self-renewal activity in CSCs of various human lung cancer cells.

### 3.5. Stemness Transcription Factors Downregulated by TDB

**Figure 6:**
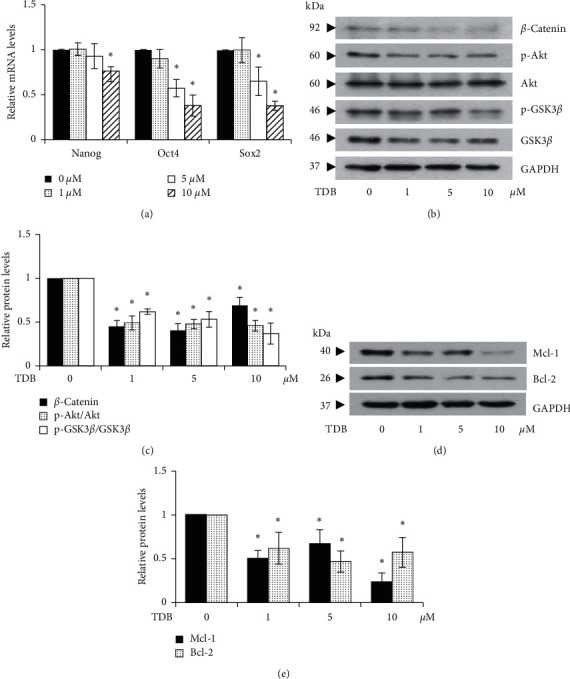
Alteration of stemness transcription factors and related proteins in human lung CSC-enriched spheroids treated with TDB. (a) RT-qPCR revealed a significant decrease in the mRNA levels of Nanog, Oct4, and Sox2 transcription factors in CSC-enriched H460 spheroids treated with TDB at 5–10 *μ*M. (b) Western blotting demonstrated the marked downregulation of p-Akt and (c) related upstream molecules including p-GSK3*β*/GSK3*β* and *β*-catenin in the spheroids after treatment of 1–10 *μ*M TDB for 24 h. (d, e) Correspondingly, expression levels of Mcl-1 and Bcl-2, antiapoptosis proteins regulated by Akt signaling, were attenuated in TDB-treated CSC spheroids. Values are means of the independent triplicate experiments ± SD. ^*∗*^*p* < 0.05 versus nontreated spheroids.

The transcription factors, Nanog, Oct4, and Sox2, are recognized as key mediators of CSC characteristics including pluripotency and self-renewal [44]. To investigate the modulatory effect on these transcription factors, mRNA levels of Nanog, Oct4, and Sox2 were detected by RT-qPCR in CSC-enriched H460 spheroids cultured with TDB (1–10 *μ*M) for 24 h. As depicted in Figure 6(a), there was no difference in the relative mRNA levels of Nanog, Oct4, and Sox2 between the spheroids treated with 1 *μ*M of TDB and untreated control. Intriguingly, the downregulation of these three transcription factors was remarkably detected in the treatment of 5–10 *μ*M TDB. This finding indicates that the suppressive effect of TDB on stemness features in human lung cancer cells is associated with decreased levels of self-renewal transcription factors including Nanog, Oct4, and Sox2.

### 3.6. TDB Modulates Akt/GSK3*β*/*β*-Catenin Signaling in Lung CSC-Enriched Spheroids

To elucidate the underlying mechanisms of TDB related to cancer stemness, the expression of upstream regulatory proteins was investigated by Western blot analysis in CSC-enriched spheroids of human lung cancer cells. After culture with 5–10 *μ*M of TDB for 24 h, there was marked diminution of expression levels of phosphorylated Akt (p-Akt), phosphorylated GSK3*β* (p-GSK3*β*), and *β*-catenin in CSC-enriched H460 spheroids (Figure 6(b)). Despite producing no significant alteration of downstream transcription factors, treatment with 1 *μ*M TDB was observed to modulate Akt/GSK3*β*/*β*-catenin signaling in CSC secondary spheroids (Figure 6(c)).

As the previous study revealed that TDB modulates Bcl-2 family proteins in human lung cancer cells via mediating Akt signaling [45], the alteration of antiapoptosis Mcl-1 and Bcl-2 proteins was additionally examined in CSC-enriched spheroids treated with TDB. The significant reduction of Mcl-1 and Bcl-2 detected via Western blot analysis was observed in CSC-enriched H460 spheroids incubated with 1–10 *μ*M TDB for 24 h (Figures 6(d) and 6(e)). These results strongly indicate the anticancer activity of TDB in targeting CSC populations of human lung cancer cells.

### 3.7. TDB Suppresses CSC Phenotype in Human Lung Cancer Cells Dependently on Akt Mediation

To further verify the underlying machinery involved in the CSC-targeted effect of TDB on human lung cancer cells, Akt, an upstream regulatory molecule, was subjected to knockdown in lung cancer H460 cells using specific siRNA.

**Figure 7:**
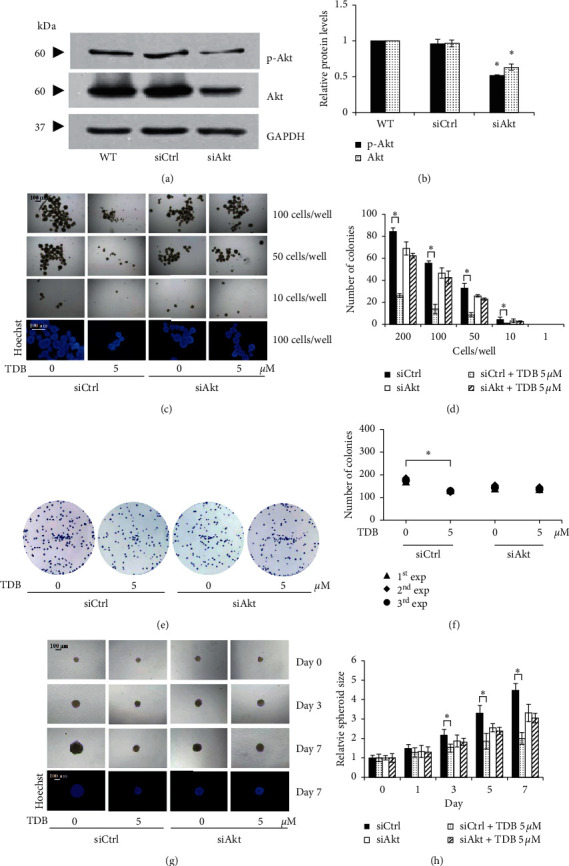
The effect of TDB in Akt knockdown human lung cancer cells. (a) The alteration of Akt and p-Akt expression levels was determined via western blot analysis in lung cancer H460 cells transfected with either small interfering RNA (siRNA) specific to human Akt (siAkt) or si-mismatch control (siCtrl) for 48 h. (b) Transfection with siAkt significantly downregulated Akt and p-Akt protein expression levels in H460 cells compared with both nontransfected wild-type (WT) cells and siCtrl-transfected cells. (c) Tumor-initiating activity of H460 transfected with either siCtrl or siAkt was evaluated in LDA assay. Photographs of tumor colonies were obtained through optical microscopy (4x), while images with bright blue fluorescence of Hoechst33342 were obtained by fluorescence microscopy (10x) after incubation for 14 days. (d) The number of tumor-initiating cells in siCtrl-transfected H460 cells was significantly decreased after treatment with 5 *μ*M TDB for 14 days, while no alteration of the colony number was observed in TDB-treated H460 cells transfected with siAkt compared with the nontreated control cells. (e) The suppressive effect on colony formation under attached condition was also determined by the clonogenic assay after culture of siRNA-transfected cells with TDB for 24 h. (f) The number of formed colonies in response to TDB (5 *μ*M) treatment was diminished only in siCtrl-transfected H460 cells, but not in siAkt-transfected cells compared with the nontreated groups. (g) Self-renewal capacity of siCtrl-transfected lung cancer cells and Akt knockdown H460 cells was depicted with the enlargement of CSC-enriched spheroids cultured for 0–7 days under detachment condition. CSC spheroids were observed under optical microscopy (10x), and images reflecting the bright blue fluorescence of Hoechst33342 were taken by fluorescence microscopy (10x). (h) Treatment with 5 *μ*M of TDB caused no alteration of the relative size of CSC spheroids derived from siAkt-transfected H460 cells, though the lowered relative spheroid size was indicated in TDB-treated siCtrl-transfected cells when compared with the untreated spheroids. Values are means of the independent triplicate experiments ± SD. ^*∗*^*p* < 0.05 versus nontreatment of each cell.

After 48 h of transfection, the significant downregulation of Akt and p-Akt protein levels was demonstrated via Western blot analysis in lung cancer H460 cells transfected with siAkt compared with both wild-type (WT) cells and si-mismatch control- (siCtrl-) transfected cells (Figure 7(a)). It is worth noting that there was no alteration of Akt and p-Akt expression level in siCtrl-transfected H460 cells compared with nontransfected wild-type cells (Figure 7(b)). Likewise, the incubation with 5 *μ*M TDB obviously suppressed tumor-initiating activity (Figure 7(c)), colony formation (Figure 7(e)), and enlargement of CSC-enriched spheroids in siCtrl-transfected H460 cells (Figure 7(g)). Surprisingly, the reduced Akt and p-Akt expression level efficiently restrained CSC-targeted anticancer activity of TDB as evidenced with no significant alteration of tumor-initiating cells (Figure 7(d)), number of formed colonies under attachment condition (Figure 7(f)), and relative CSC spheroid size in siAkt-transfected H460 cells culture with 5 *μ*M TDB (Figure 7(h)). These results suggest that TDB suppression of CSC phenotype in human lung cancer cells may partially be due to the Akt-dependent mechanism.

## 4. Discussion

CSCs, the rare subpopulations in tumor tissue, are characterized by stemness features of self-renewal and pluripotency, which contribute to tumorigenicity, chemotherapeutic resistance, and ultimately cancer relapse [46]. Thus, CSC-targeted therapy is of interest as a novel and promising strategy for cancer treatment evidenced by the success of CSC biomarkers-mediated drug delivery systems in both preclinical and clinical studies [47–49]. However, the key limitation of conventional chemotherapies is that these agents primarily target proliferative tumor bulk, but not tumor-initiating cells [46, 48]. Natural products are widely recognized as sources of drug leads, especially in cancer therapy [50]. Garnering much research attention, natural compounds were recently reported to produce a suppressive effect on CSC phenotype and related regulatory pathways [24, 32, 51]. Due to the crucial role of CSCs in initiation and maintenance of lung cancer [52], the results herein revealing anticancer activity of TDB, a bibenzyl extracted from *D. ellipsophyllum*, against lung CSCs could guide the future investigations and innovations related to CSC-targeted therapy.

Tumorigenesis and self-renewal of various CSCs have been reported to be mediated by Akt/GSK3*β*/*β*-catenin pathway [53]. Activated Akt (p-Akt), a recognized upstream molecule triggering survival cascade, is likewise linked to the Wnt/*β*-catenin signaling, which also regulates CSC features. Phosphorylation at Ser 9 of GSK3*β* by p-Akt results in deactivation of GSK3*β*, which in turn stabilizes *β*-catenin to stimulate the expression of stemness-related genes [54]. Recently, the induction of Wnt/*β*-catenin signaling via PI3K/Akt/GSK3*β* cascade has been proposed to drive drug resistance in cancer cells [55]. *In vivo* evidence suggests that Akt-driven CSC enrichment is mediated by activation of the Wnt/*β*-catenin pathway through GSK3*β* phosphorylation [56]. It has been reported that tobacco smoke, an important risk factor for lung cancer, stimulates the Wnt/*β*-catenin pathway and enhances the CSC phenotype in lung cancer patients [52]. Interestingly, small molecules derived from natural products are deemed to possess much therapeutic potential and were even observed to inhibit CSC subpopulations and abolish tumor growth *in vivo* through inhibition of the Wnt/*β*-catenin pathway [57, 58]. These reports correlate with the observed downregulation of p-Akt along with the reduced levels of p-GSK3*β* (Ser 9) and *β*-catenin in TDB-treated CSC-enriched spheroids (Figures 6(b) and 6(c)). Additionally, mTOR signaling, which is widely implicated in cancer pathology, mediates CSC formation and drug resistance through phosphorylated Akt at Ser 473 [59]. The revealed downregulation of p-Akt (Ser 473) in CSC-enriched spheroids treated with TDB indicates some molecular insight into the lung CSC-targeted capacity of TDB.

The effects of TDB on initiation of tumor growth and CSC self-renewal were revealed via clonogenic assay under both detached and attached conditions [35, 36] and the 3D spheroid assay, the latter particularly approximating *in vivo* conditions and serving as a robust *in vitro* model for screening of anticancer compounds [60]. Under the detached condition, characteristics and cellular signals involved in the stem-like phenotype of 3D cancer spheroids considerably resemble *in vivo* solid tumor [42, 60]. While animal studies will further validate antitumorigenic efficacy, the suppression of tumor initiation (Figures 2 and 3) and self-renewal of CSCs (Figures 4 and 5) in various human lung cancer cells, namely, H460 (p53 and KRas wild type), H23 (p53 and KRas mutant), and A549 (KRas mutant), markedly observed after treatment with TDB (5–10 *μ*M) strongly support the CSC-targeted potential of this bibenzyl compound.

The suppressive activity on CSC phenotypes correlates with the diminished expression of Oct4, Sox2, and Nanog, the downstream transcription factors of *β*-catenin, in TDB-treated CSC spheroids (Figure 6(a)). Oct4, Sox2, and Nanog are recognized transcription factors mediating tumor transformation, tumorigenicity, and metastasis. These transcription factors play crucial roles in sustained properties of self-renewal and pluripotency in embryonic stem cells [61]. The complex network governing coupregulation of Oct4, Sox2, and Nanog is also noted in many human CSCs derived from renal cell carcinoma [62], hepatocellular carcinoma [63], breast cancer [64], and lung cancer [26]. Recently, the suppression of CSC phenotype in lung cancer induced by alpha-lipoic acid was posited to be driven by the reduction of p-Akt level, which led to the depletion of p-GSK3*β*, *β*-catenin, and Oct4 [25]. Consistent with this finding, the diminution of self-renewal and tumor-initiating activity in lung CSCs cultured with TDB may also result from downregulation of Oct4, Sox2, and Nanog mediated through modulation of Akt/Gsk3*β*/*β*-catenin signal. Additionally, the loss of inhibitory effect of TDB on self-renewal and tumor initiation in Akt knockdown lung cancer cells (Figure 7) further verifies the Akt-dependent mechanism of TDB.

TDB, a bibenzyl extracted from *D. ellipsophyllum*, has been previously recognized for potent cytotoxicity [45], potential antimetastasis activity, and EMT suppression capacity in human lung cancer cells [65]. Furthermore, TDB presents a good safety and efficacy profile, which is evidenced by the lower IC_50_ reported in various human lung cancer cells compared with normal cells (Figure 1(c)) and the alteration of apoptosis-regulating proteins leading to selective apoptosis induction in lung cancer cells treated with TDB [45]. It has been well-established that Akt also plays a pivotal role in apoptosis through the modulation of Bcl-2 family proteins [66, 67]. The upregulation of Bcl-2 has been reported to promote cell survival and chemotherapeutic resistance in lung cancer cells isolated from patients. Moreover, the antiapoptosis Mcl-1 protein is also known to mediate the preservation of stemness features [68, 69]. The initiation, growth, and progression of cancer lesions have been promoted by Akt-mediated Mcl-1 expression [59]. Cotargeting of Bcl-2 and Mcl-1 is thought to improve mitochondrial priming towards apoptosis, which could lead to better therapeutic responses [70]. The decreased expression levels of Bcl-2 and Mcl-1 in TDB-treated CSC-enriched spheroids (Figure 6(e)) and modulation of stemness-related pathways strongly suggest the promising anticancer activity of TDB particularly targeting lung CSCs.

## 5. Conclusion

**Figure 8:**
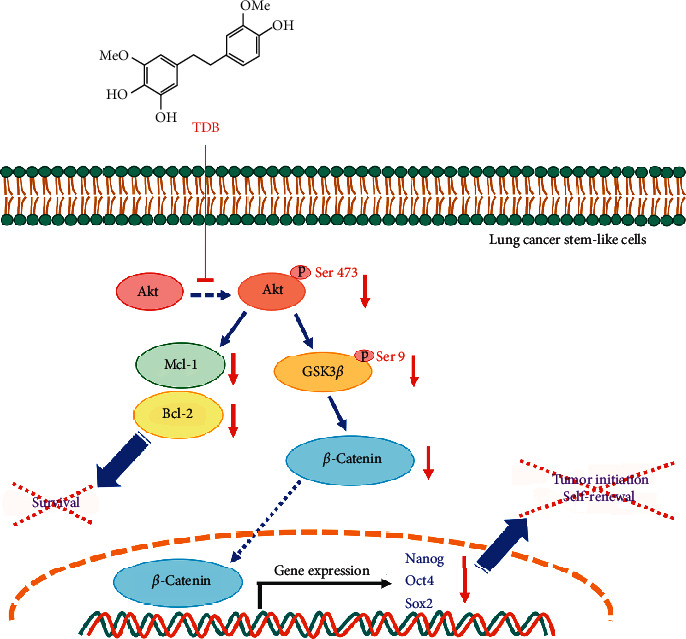
The proposed regulatory mechanism of TDB related to the suppressive effect on lung cancer stem-like cells (CSCs). The stemness features of self-renewal and tumor initiation in lung CSCs were attenuated by TDB through the modulation of Akt-mediated signals as evidenced by the downregulation of p-Akt, p-GSK3*β*, and *β*-catenin following reduced expression level of CSC transcription factors, Nanog, Oct4, and Sox2. Moreover, repression of p-Akt could lead to the diminished expression levels of antiapoptosis Bcl-2 and Mcl-1 in lung CSC-enriched populations cultured with TDB.

In the present study, the novel anticancer activity of TDB, a bibenzyl from *D. ellipsophyllum*, towards CSCs of human lung cancer cells is revealed as evidenced by the suppression of tumor initiation and self-renewal properties associated with downregulation of Akt/GSK3*β*/*β*-catenin signal and related proteins (Figure 8). The obtained information would thus support the development of TDB as an effective CSC-targeted treatment for lung cancer.

## Data Availability

The data used to support the findings of this study are included within the article and available from the corresponding author upon request.

## Abbreviations

CSC: Cancer stem-like cell
TDB: 4,5,4′-Trihydroxy-3,3′-dimethoxybibenzyl
NSCLC: Non-small-cell lung cancer
Oct4: Octamer-binding transcription factor 4
Sox2: Sex-determining region Y-box 2
ESC: Embryonic stem cell
PI3K: Phosphoinositide 3-kinases
Akt: Protein kinase B
GSK3*β*: Glycogen synthase kinase 3*β*
PI: Propidium iodide
MTT: 3-(4,5-Dimethylthiazol-2-yl)-2,5-diphenyltetrazolium bromide
DMSO: Dimethyl sulfoxide
Bcl-2: B-cell lymphoma 2
Mcl-1: Myeloid cell leukemia 1
GAPDH: Glyceraldehyde 3-phosphate dehydrogenase
HRP: Horseradish peroxidase
RPMI: Roswell Park Memorial Institute
DMEM: Dulbecco's Modified Eagle Medium
FBS: Fetal bovine serum
PBS: Phosphate-buffered saline
3D: Three-dimensional
LDA: Limiting dilution assay
RIPA: Radioimmunoprecipitation assay lysis buffer
BCA: Bicinchoninic acid protein assay kit
SDS-PAGE: Sodium dodecyl sulfate-polyacrylamide gel electrophoresis
TBST: Tris-buffered saline plus Tween 20.
